# Positron Emission Tomography Imaging of CD105 Expression with a ^64^Cu-Labeled Monoclonal Antibody: NOTA Is Superior to DOTA

**DOI:** 10.1371/journal.pone.0028005

**Published:** 2011-12-09

**Authors:** Yin Zhang, Hao Hong, Jonathan W. Engle, Jero Bean, Yunan Yang, Bryan R. Leigh, Todd E. Barnhart, Weibo Cai

**Affiliations:** 1 Department of Medical Physics, University of Wisconsin - Madison, Madison, Wisconsin, United States of America; 2 Department of Radiology, University of Wisconsin - Madison, Madison, Wisconsin, United States of America; 3 TRACON Pharmaceuticals, Inc., San Diego, California, United States of America; 4 University of Wisconsin Carbone Cancer Center, Madison, Wisconsin, United States of America; Carl-Gustav Carus Technical University-Dresden, Germany

## Abstract

Optimizing the in vivo stability of positron emission tomography (PET) tracers is of critical importance to cancer diagnosis. In the case of ^64^Cu-labeled monoclonal antibodies (mAb), in vivo behavior and biodistribution is critically dependent on the performance of the bifunctional chelator used to conjugate the mAb to the radiolabel. This study compared the in vivo characteristics of ^64^Cu-labeled TRC105 (a chimeric mAb that binds to both human and murine CD105), through two commonly used chelators: 1,4,7-triazacyclononane-1,4,7-triacetic acid (NOTA) and 1,4,7,10-tetraazacyclododecane-1,4,7,10-tetraacetic acid (DOTA). Flow cytometry analysis confirmed that chelator conjugation of TRC105 did not affect its CD105 binding affinity or specificity. PET imaging and biodistribution studies in 4T1 murine breast tumor-bearing mice revealed that ^64^Cu-NOTA-TRC105 exhibited better stability than ^64^Cu-DOTA-TRC105 in vivo, which resulted in significantly lower liver uptake without compromising the tumor targeting efficiency. In conclusion, this study confirmed that NOTA is a superior chelator to DOTA for PET imaging with ^64^Cu-labeled TRC105.

## Introduction

Radiolabeled antibodies have been used in the clinic for diagnostic and therapeutic applications for over 40 years [1], [2]. Monoclonal antibody (mAb)-based positron emission tomography (PET), termed “immunoPET”, is an attractive method for non-invasive tumor detection since this strategy combines the high sensitivity of PET with the high antigen specificity of mAbs. If the mAb is used for systemic therapy of cancer, either as a single agent or in combination with other anti-cancer drugs, immunoPET with the radiolabeled mAb can be used for tumor detection, patient selection, as well as treatment planning [3].

Commonly used PET isotopes for antibody labeling include ^64^Cu (t_1/2_ = 12.7 h), ^86^Y (t_1/2_ = 14.7 h), ^89^Zr (t_1/2_ = 3.3 d), ^124^I (t_1/2_ = 4.2 d), among others [4]. Currently, ^64^Cu is the most widely used isotope for immunoPET, partly due to its wide availability, low cost, and versatile chemistry. The E_max_ of 656 keV for its positron emission, which is comparable to that of ^18^F and lower than that of ^124^I, can produce PET images with good spatial resolution. In addition, there are several radioisotopes of Cu available, which allow for both diagnostic imaging (with ^60/61/62/64^Cu) and therapeutic applications (with ^64/67^Cu) [5].

One of the key requirements for accurate PET imaging with ^64^Cu-labeled mAbs is that the tracer should be sufficiently stable during the imaging period, since PET scanners detect the distribution of ^64^Cu instead of the mAb itself. Over the years, many bifunctional chelators have been investigated for ^64^Cu-labeling, such as 1,4,7,10-tetraazacyclododecane-1,4,7,10-tetraacetic acid (DOTA) [6], [7], 1,4,7-triazacyclononane-1,4,7-triacetic acid (NOTA) [8]–[11], 1,4,8,11-tetraazacyclotetradecane-1,4,8,11-tetraacetic acid (TETA) [12], 1,4,8,11-tetraazacyclotetradecane-N,N′,N″,N‴-tetraacetic acid (BAT) [13], 4,11-bis(carboxymethyl)-1,4,8,11-tetraazabicyclo[6.6.2]hexadecane (CB-TE2A) [14], [15], 1,8-diamino-3,6,10,13,16,19-hexaazabicyclo(6,6,6)eicosane (Diamsar) [16], [17], among many others [4], [18].

CD105 (endoglin), a 180 kDa disulfide-linked homodimeric transmembrane protein, is one of the most suitable markers for tumor angiogenesis [19], [20]. In contrast to CD31, which is expressed on both normal and proliferating vasculature, CD105 is only over-expressed on proliferating tumor endothelial cells and CD105 immunohistochemistry (IHC) is the accepted standard for detecting proliferating vessels (i.e. neovessels) within tumors. Not surprisingly, high microvessel density (MVD) of CD105-expressing vessels correlates with poor prognosis and/or survival in more than 10 solid tumor types [19], [21]. TRC105 is a human/murine chimeric IgG1 mAb which binds to both human and murine CD105 [22]. The murine parent antibody of TRC105 (SN6j) has demonstrated anti-cancer efficacy in animal tumor models [20]. Recently, a multicenter Phase 1 first-in-human dose-escalation trial of TRC105 was completed in the United States and Phase 2 therapy trials are underway in ovarian, prostate, bladder, liver, and breast cancer [23].

We recently reported the first PET imaging of CD105 expression using ^64^Cu-DOTA-TRC105 in 4T1 murine breast tumor-bearing mice [24]. Prominent and persistent tracer uptake in the 4T1 tumor was observed. In addition, blocking experiments with unlabeled TRC105, control studies with ^64^Cu-DOTA-cetuximab (an isotype matched chimeric mAb that binds to the human epidermal growth factor receptor [25], [26]), as well as ex vivo histology all confirmed the in vivo target specificity of ^64^Cu-DOTA-TRC105. In order to further improve the in vivo behavior of ^64^Cu-labeled TRC105, this study investigated the performance of TRC105 conjugated to ^64^Cu using a NOTA chelator (i.e. ^64^Cu-NOTA-TRC105) through PET imaging and biodistribution studies. Through direct comparison with data obtained from our previous study of ^64^Cu-DOTA-TRC105 [24], we evaluated the effect of bifunctional chelators (i.e. DOTA and NOTA) on the in vivo behavior of the PET tracers.

## Results

### In Vitro Investigation of NOTA-TRC105

**Figure 1:**
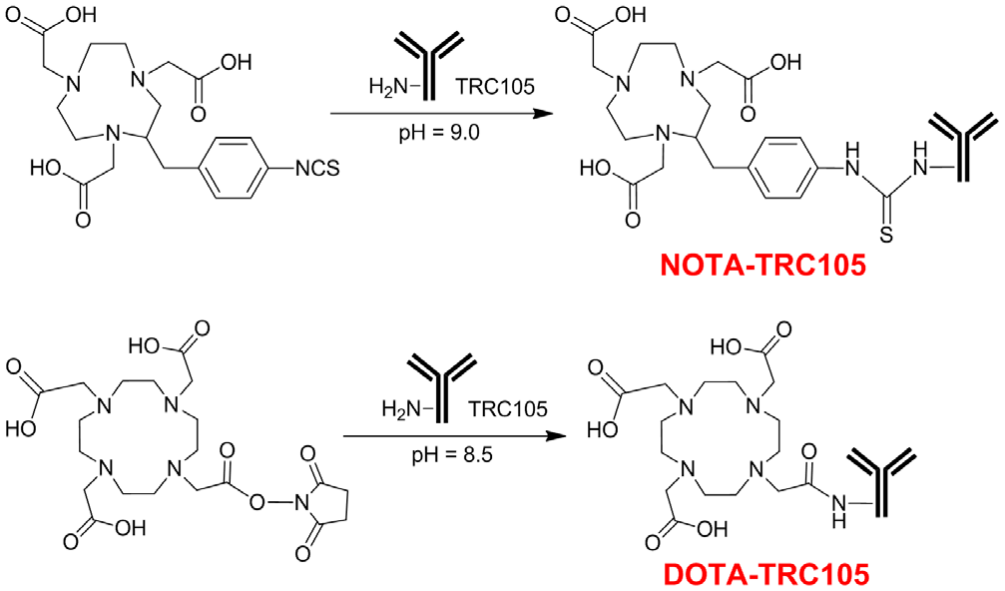
NOTA and DOTA conjugation of TRC105.

**Figure 2:**
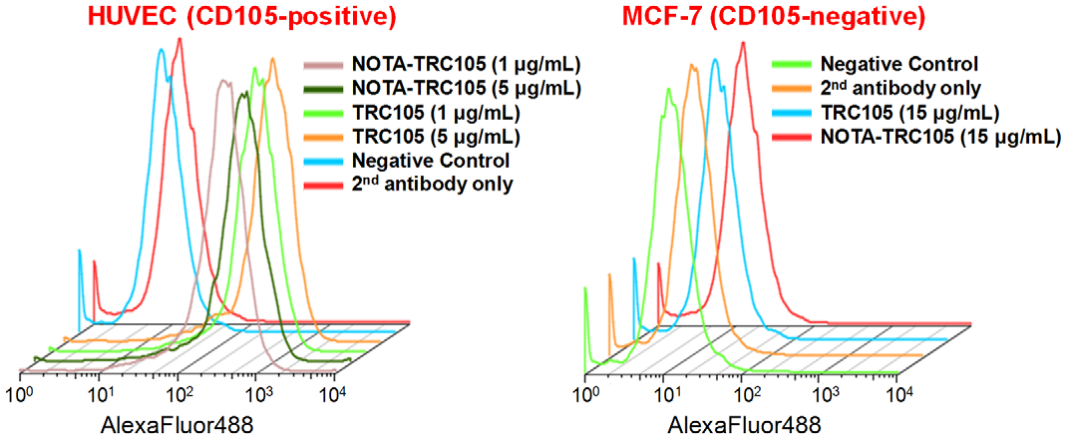
Flow cytometry analysis of TRC105 and NOTA-TRC105 in HUVECs (CD105-positive) and MCF-7 (CD105-negative) cells at different concentrations.

The chemistry for NOTA and DOTA conjugation to TRC105 was similar (**Figure 1**). NOTA conjugation of TRC105 did not alter its CD105 binding affinity, as evidenced by fluorescence-activated cell sorting (FACS) analysis (**Figure 2**). At non-antigen-saturating conditions, FACS analysis of human umbilical vein endothelial cells (HUVECs, CD105-positive [24]) revealed no observable difference between TRC105 and NOTA-TRC105 at 1 µg/mL or 5 µg/mL. The binding to HUVECs was CD105-specific, as neither TRC105 nor NOTA-TRC105 bound to CD105-negative MCF-7 cells. These data were comparable to our previous studies using DOTA-TRC105 [24], with neither NOTA or DOTA conjugation affecting the antigen binding avidity or specificity of TRC105.

### ^64^Cu-Labeling

^64^Cu-labeling including final purification using PD-10 columns took 80±10 min (n = 5). The decay-corrected radiochemical yield was >85% based on 25 µg of TRC105 (DOTA-TRC105 or NOTA-TRC105) per 37 MBq of ^64^Cu, and the radiochemical purity was >98%. The specific activity of ^64^Cu-NOTA-TRC105 was about 3.0 GBq/mg protein, virtually identical to that of ^64^Cu-DOTA-TRC105 [24], assuming complete recovery of NOTA-TRC105 after size exclusion chromatography. Based on the specific activity of ^64^Cu (∼5% of the theoretical value of 254 Ci/µmol) and the amount of NOTA-TRC105 used for radiolabeling, it was calculated that there were 0.42±0.04 (n = 3) ^64^Cu ion per molecule of TRC105.

### Small Animal PET Studies

**Figure 3:**
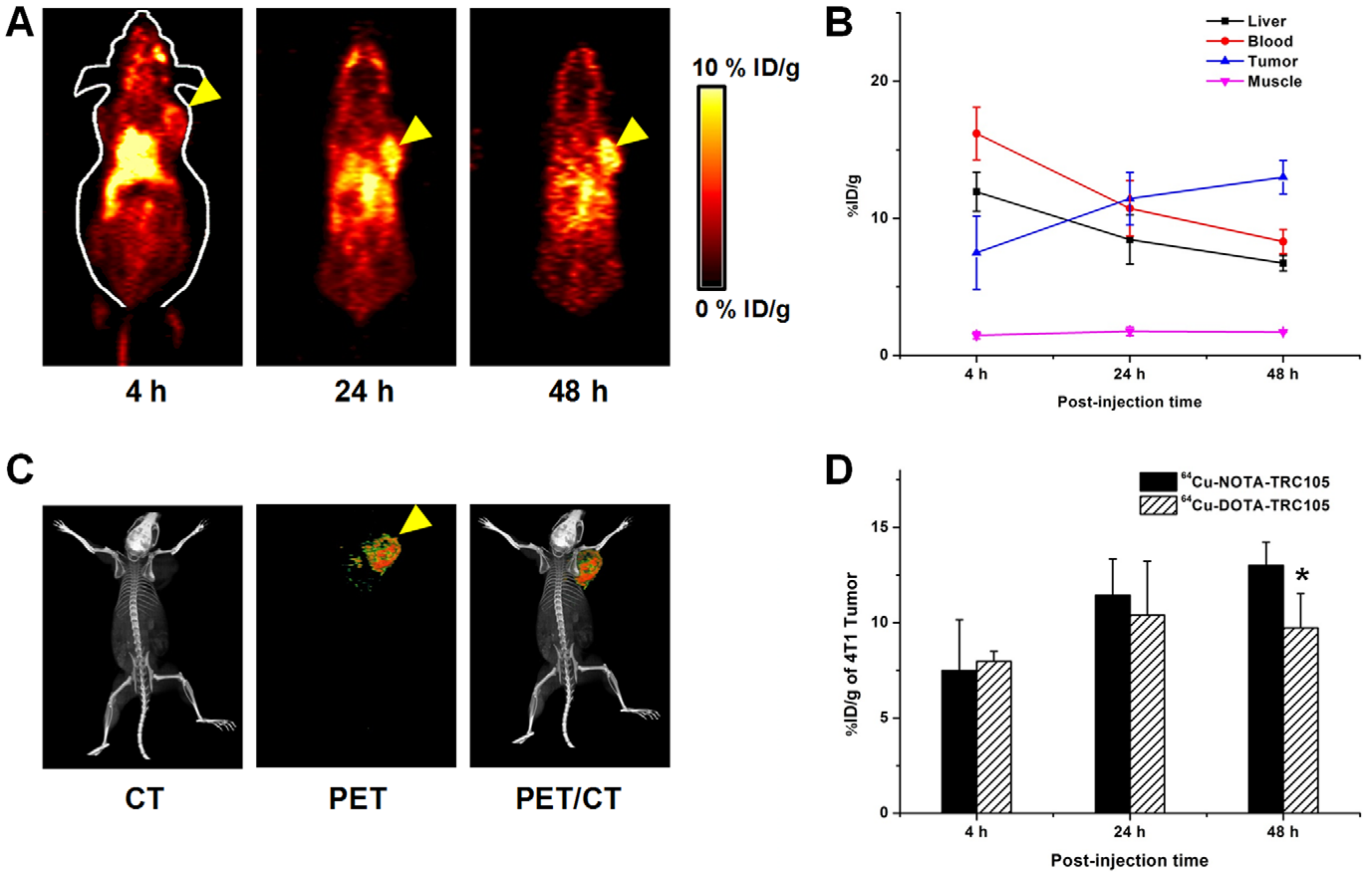
Small animal PET imaging of 4T1 tumor-bearing mice. **A.** Serial coronal PET images at 4, 24, and 48 h post-injection of ^64^Cu-NOTA-TRC105. Tumors are indicated by arrowheads. **B.** Time-activity curves of tumor, liver, blood, and muscle after intravenous injection of ^64^Cu-NOTA-TRC105 into 4T1 tumor-bearing mice (n = 3) **C.** Representative PET/CT images of ^64^Cu-NOTA-TRC105 in 4T1 tumor-bearing mice at 48 h post-injection. **D.** Comparison of the 4T1 tumor uptake of ^64^Cu-NOTA-TRC105 and ^64^Cu-DOTA-TRC105. *: P<0.05.

The time points of 4, 24, and 48 h post-injection (p.i.) were chosen for serial PET scans after intravenous tracer injection, based on our previous studies with ^64^Cu-DOTA-TRC105 and other ^64^Cu-labeled mAbs [6], [24], [25], [27]. Coronal slices containing the 4T1 tumor are shown in **Figure 3A** and representative PET/CT fused images of a mouse at 48 h p.i. of ^64^Cu-NOTA-TRC105 are shown in **Figure 3C**. The quantitative data obtained from ROI analysis are shown in **Figure 3B&D**. Consistent with our previous findings of radiolabeled antibodies, the uptake of ^64^Cu-NOTA-TRC105 in the liver (due to hepatic clearance and potential trans-chelation of ^64^Cu) and blood pool (due to long circulation half-life of the antibody) was prominent at early time points and gradually declined over time. Liver uptake of ^64^Cu-NOTA-TRC105 was 11.9±1.4, 8.5±1.8, and 6.7±0.6 percentage injected dose per gram of tissue (%ID/g) at 4, 24, and 48 h p.i. respectively, while the radioactivity in the blood was 16.2±1.9, 10.7±2.0, and 8.3±0.9%ID/g at 4, 24, and 48 h p.i. respectively (n = 3; **Figure 3B**). Uptake of ^64^Cu-NOTA-TRC105 in the 4T1 tumor was clearly visible at 4 h p.i. and plateaued after 24 h p.i. (7.5±2.7, 11.4±1.9, and 13.0±1.2%ID/g at 4, 24, and 48 h p.i. respectively; n = 3; **Figure 3B&D**), indicating antigen specific binding.

Comparing the two TRC105-based tracers, tumor uptake of ^64^Cu-NOTA-TRC105 increased over time while that of ^64^Cu-DOTA-TRC105 peaked at 24 h p.i. and slightly decreased at 48 h p.i. At the 48 h time point, the difference between tumor uptake of the two tracers was statistically significant (P<0.05; n = 3; **Figure 3D**).

### Biodistribution Studies

**Figure 4:**
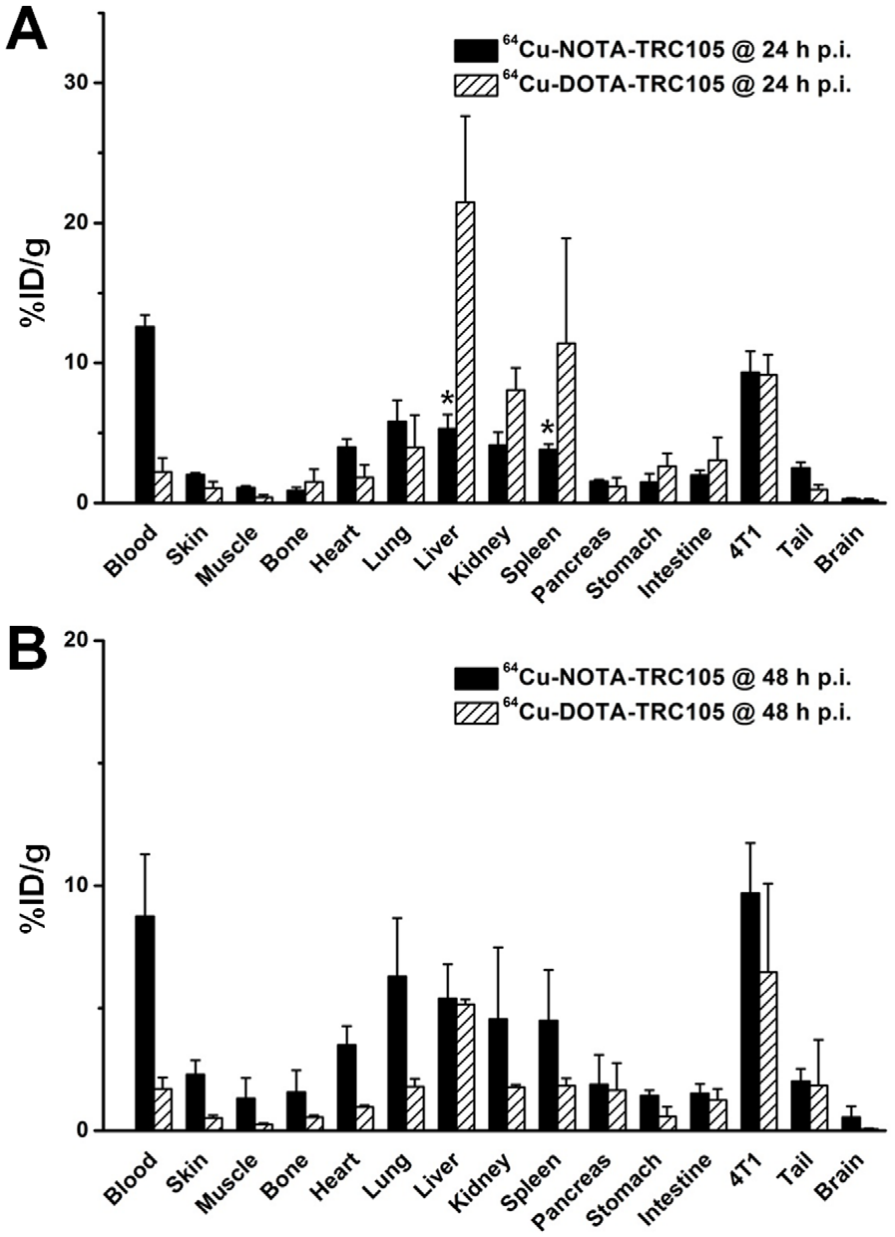
Biodistribution studies in 4T1 tumor-bearing mice. **A.** Biodistribution of ^64^Cu-NOTA-TRC105 and ^64^Cu-DOTA-TRC105 at 24 h post-injection (n = 3). **B.** Biodistribution of ^64^Cu-NOTA-TRC105 and ^64^Cu-DOTA-TRC105 at 48 h post-injection (n = 3). *: P<0.05.

Following the terminal PET scans at 48 h p.i., all mice were euthanized for biodistribution studies. In addition, a separate group of three mice were injected with ^64^Cu-NOTA-TRC105 and euthanized at 24 h p.i. for biodistribution studies (**Figure 4A**). The uptake of ^64^Cu-NOTA-TRC105 in the liver and spleen was significantly lower than that of ^64^Cu-DOTA-TRC105 at 24 h p.i., while the radioactivity in the blood pool showed an opposite trend, suggesting significantly better in vivo stability and prolonged circulation half-life of ^64^Cu-NOTA-TRC105. Uptake of ^64^Cu-NOTA-TRC105 in all tissues (except blood) was lower than that in the 4T1 tumor at 24 h p.i. However, the 4T1 tumor uptake of the two tracers was similar at 24 h p.i., based on both region-of-interest (ROI) analysis of PET data and biodistribution results.

Comparing the biodistribution of ^64^Cu-NOTA-TRC105 and ^64^Cu-DOTA-TRC105 at 48 h p.i. (**Figure 4B**), blood radioactivity was still prominent for ^64^Cu-NOTA-TRC105, which was significantly higher than that of ^64^Cu-DOTA-TRC105. The 4T1 tumor uptake of ^64^Cu-NOTA-TRC105 was also higher than that of ^64^Cu-DOTA-TRC105 at 48 h p.i., suggesting higher stability of ^64^Cu-NOTA-TRC105 than ^64^Cu-DOTA-TRC105. Interestingly, the uptake of ^64^Cu-DOTA-TRC105 dropped significantly in all major organs from 24 h p.i. to 48 h p.i., while the uptake of ^64^Cu-NOTA-TRC105 remained stable in all organs. This phenomenon may be attributed to the longer circulation half-life of ^64^Cu-NOTA-TRC105, which resulted in sufficient amount of the tracer circulating in the blood available for antigen binding, whereas ^64^Cu-DOTA-TRC105 was gradually cleared with little surplus tracer available within the circulation. In addition, the difference in stability of ^64^Cu-NOTA and ^64^Cu-DOTA complexes certainly played a role.

At both 24 h and 48 h p.i., tumor uptake of ^64^Cu-NOTA-TRC105 was higher than all major organs in the mice, thus providing excellent tumor contrast (**Figure 3** **&** **4**). For ^64^Cu-DOTA-TRC105, such tumor contrast was only achieved at 48 h p.i., after the majority of initial liver and spleen uptake of the tracer cleared over time. Overall, the quantification results obtained from biodistribution studies and PET scans matched very well, confirming that quantitative ROI analysis of non-invasive microPET scans accurately reflected the distribution of PET tracers in vivo.

## Discussion

In vivo stability of ^64^Cu-chelates has been hotly debated over the last decade, and many chelators have been designed and evaluated for ^64^Cu-labeling of various targeting ligands [4]. Based on available literature data, the in vitro thermodynamic stability constants of Cu-DOTA and Cu-NOTA are similar at 25°C [28], [29]. However, these constants were measured in a simple in vitro chemical system, which does not represent the biological environment in vivo. Furthermore, attaching a mAb to the chelator will significantly improve the stability of the ^64^Cu-chelator complex, since trans-chelation of Cu requires conformational change of the chelator, which is much more difficult when a mAb is attached. In this study, we evaluated the in vivo behavior of two PET tracers, ^64^Cu-DOTA-TRC105 and ^64^Cu-NOTA-TRC105, and compared the effect of the bifunctional chelator on ^64^Cu/tracer biodistribution.

The two conjugates showed similar tumor targeting efficiency at 24 h p.i., indicating that different chelators do not significantly impact tracer uptake in the tumor. However, tracer uptake in many normal organs exhibited significant differences, including the liver, spleen, and kidneys. Since liver is one of the major organs for ^64^Cu trans-chelation [30], liver uptake of ^64^Cu-DOTA-TRC105 was much higher than that of ^64^Cu-NOTA-TRC105 at 24 h p.i. Lung uptake of ^64^Cu-NOTA-TRC105 was also much higher than that of ^64^Cu-DOTA-TRC105 at 48 h p.i., which is likely due to the prolonged circulation half-life of ^64^Cu-NOTA-TRC105 (as the lung is a very well-perfused organ). Although tumor uptake of the two tracers was similar at 24 h p.i., uptake of ^64^Cu-DOTA-TRC105 dropped slightly from 24 h to 48 h p.i. while the uptake of ^64^Cu-NOTA-TRC105 remained relatively stable. One possible explanation for this finding is the acidic environment in the tumor tissue. It has been suggested that the kinetic stability of a Cu complex is a function of the change of its degree of protonation as a response to changes in pH, and Cu-NOTA was found to be more stable than Cu-DOTA at pH 4.6 [11]. Although the tumor pH value may not be as low as 4.6 in many cases, the typically acidic microenvironment of the tumor [31] may be one cause for a decrease in tumor uptake of ^64^Cu-DOTA-TRC105 over time.

Recently, an elegant study compared the effect of several bifunctional chelators on the biodistribution of a ^64^Cu-labeled antibody [11], which concluded that thermodynamic stability of ^64^Cu-chelator complexes did not significantly influence tumor uptake of the tracer. However, there were significant differences in tracer concentration in other tissues, including those involved in clearance of the radioimmunoconjugate (e.g., liver and spleen). Our findings are in agreement with these data, as tumor uptake of the two tracers was similar while the liver/spleen uptake was quite different for ^64^Cu-NOTA-TRC105 and ^64^Cu-DOTA-TRC105. Taken together the findings from these two studies and various other literature reports, we conclude that NOTA is one of the best chelators for ^64^Cu-labeling of macromolecules, proteins, or nanomaterials [8]–[10], [32].

One key challenge in antibody labeling is to minimize the potential interference with its antigen binding affinity/specificity. There is only one lysine residue in each of the complementarity-determining regions (CDRs) of TRC105 [20], which has a total of ∼1400 amino acid residues and ∼70 lysines. Therefore, the possibility of DOTA/NOTA conjugation at the lysine residue within the CDR is extremely low, which was confirmed by FACS analysis at several non-antigen-saturating conditions (**Figure 2**). To confirm that tumor uptake of TRC105-based imaging probes measured by non-invasive imaging techniques was indeed CD105 specific, various control experiments (e.g. blocking study with unconjuagted TRC105, as well as the use of cetuximab as an isotype-matched control), in vitro/ex vivo studies (e.g. FACS and histological analysis), as well as biodistribution and ex vivo imaging studies were performed for validation purposes [24], [33], [34].

The primary focus of this study was to compare ^64^Cu-labeled TRC105 through DOTA and NOTA, and we found that NOTA was superior to DOTA in many aspects as detailed above. However, even though the in vivo kinetic stability of Cu-DOTA is lower than Cu-NOTA, DOTA is a universal chelator which can complex a wide variety of both imaging and therapeutic radioisotopes. The same DOTA-mAb conjugate can therefore be employed for both imaging and therapeutic applications, with the use of appropriate isotopes, without altering its pharmacokinetics and tumor targeting efficacy. With future clinical translation and cGMP (current good manufacturing practice) compliance in mind, using the same mAb conjugate for both imaging and therapeutic applications (i.e. “theranostics”) is undoubtedly more advantageous than using two different conjugates. If the major goal of a future study is to reduce the radiation dose to the liver, NOTA is the chelator of choice for ^64^Cu. If the same antibody will be used for radioimmunotherapy applications (e.g. with ^90^Y and/or ^177^Lu), DOTA may be a better option.

In conclusion, we investigated the properties of ^64^Cu-NOTA-TRC105 both in vitro and in vivo. Compared with our previous study of ^64^Cu-DOTA-TRC105, ^64^Cu-NOTA-TRC105 was more stable in vivo which is in agreement with previous literature reports [11], [35]. We found that the nature of the bifunctional chelator affected the biodistribution of ^64^Cu-labeled mAb to some extent. However, tumor targeting efficacy was not significantly impacted by the bifunctional chelator. Since TRC105 is already in Phase 2 trials and therapeutic efficacy has been shown in various animal tumor models and certain cancer patients, clinical translation of ^64^Cu-NOTA-TRC105 is desirable where it may be used for patient selection/stratification, as well as evaluating the pharmacokinetics, tumor targeting efficacy, dose optimization, and dose interval of TRC105 and TRC105-based cancer therapeutics in the clinic.

## Materials and Methods

### Reagents

TRC105 was provided by TRACON pharmaceuticals Inc. (San Diego, CA). AlexaFluor488-conjugated secondary antibody was purchased from Jackson Immunoresearch Laboratories, Inc. (West Grove, CA). 2-S-(4-Isothiocyanatobenzyl)-1,4,7-triazacyclononane-1,4,7-triacetic acid (p-SCN-Bn-NOTA) was purchased from Macrocyclics, Inc. (Dallas, TX) and Chelex 100 resin (50–100 mesh) was purchased from Sigma-Aldrich (St. Louis, MO). Water and all buffers were of Millipore grade and pre-treated with Chelex 100 resin to ensure that the aqueous solution was heavy-metal free. PD-10 desalting columns were purchased from GE Healthcare (Piscataway, NJ). ^64^Cu was produced via a ^64^Ni(p,n)^64^Cu reaction using a cyclotron at the University of Wisconsin-Madison.

### Cell Lines and Animal Model

All animal studies were conducted under a protocol approved by the University of Wisconsin Institutional Animal Care and Use Committee (M02239). 4T1 murine breast cancer, MCF-7 human breast cancer, and HUVECs were obtained from the American Type Culture Collection (ATCC, Manassas, VA) and cultured as previously described [24], [33], [34]. Cells were used for in vitro and in vivo experiments when they reached ∼80% confluence. To generate the 4T1 tumor model, four- to five-week-old female Balb/c mice (Harlan, Indianapolis, IN) were each subcutaneously injected with 2×10^6^ cells, suspended in 100 µL of 1∶1 mixture of RPMI 1640 and matrigel (BD Biosciences, Franklin lakes, NJ) [36]. The tumor sizes were monitored every other day and mice were used for in vivo experiments when the tumor diameter reached 6–8 mm (typically less than 2 weeks after inoculation).

### NOTA Conjugation and ^64^Cu-Labeling

NOTA conjugation was carried out at pH 9.0, with the ratio of p-SCN-Bn-NOTA∶TRC105 being 25∶1, the same as that used previously for DOTA-TRC105 (**Figure 1**). NOTA-TRC105 was purified using PD-10 columns. For radiolabeling, ^64^CuCl_2_ (74 MBq) was diluted in 300 µL of 0.1 M sodium acetate buffer (pH 6.5) and added to 50 µg of NOTA-TRC105. The reaction mixture was incubated for 30 min at 40°C with constant shaking. ^64^Cu-NOTA-TRC105 was purified using PD-10 columns with phosphate-buffered saline (PBS) as the mobile phase. The radioactive fractions containing ^64^Cu-NOTA-TRC105 were collected and passed through a 0.2 µm syringe filter for in vivo experiments.

### Flow Cytometry

The immunoreactivity of TRC105 and NOTA-TRC105 to HUVECs (high CD105 expression [37], [38]) and MCF-7 (CD105-negative [38]) cells were evaluated by flow cytometry. Cells were harvested and suspended in cold PBS with 2% bovine serum albumin (BSA) at a concentration of 5×10^6^ cells/ml. These cells were incubated with TRC105 or NOTA-TRC105 (1 or 5 µg/ml) for 30 min at room temperature (RT), washed three times with cold PBS, and centrifuged at 1,000 rpm for 5 min. The cells were then incubated with AlexaFluor488-labeled goat anti-human IgG for 30 min at RT. Afterwards, the cells were washed and analyzed by flow cytometry using a BD FACSCalibur 4-color analysis cytometer equipped with 488 nm and 633 nm lasers (Becton-Dickinson, San Jose, CA) and FlowJo analysis software (Tree Star, Inc., Ashland, OR).

### PET Imaging and Biodistribution Studies

PET and PET/CT scans at various time points p.i., image reconstruction, and ROI analysis were performed using a microPET/microCT Inveon rodent model scanner (Siemens Medical Solutions USA, Inc.) as described previously [34]. Each tumor-bearing mouse was injected with 5–10 MBq of ^64^Cu-NOTA-TRC105 via tail vein and 3–15 min static PET scans were performed. Quantitative data were presented as %ID/g. Biodistribution studies were carried out after the last PET scans to validate the PET results.

### Statistical Analysis

Quantitative data were expressed as mean ± SD. Means were compared using Student's t-test. P values<0.05 were considered statistically significant.
